# The Life and Work of Prof. Dr. Antapur Venkoba Rao: The Enduring Legacy of an Innovative Mind

**DOI:** 10.7759/cureus.76306

**Published:** 2024-12-24

**Authors:** Nischal Krishna Macharla, Ramya Rachel Jetty, Aruna Kaki, Tharini D, Arul Saravanan R

**Affiliations:** 1 Psychiatry, SRM Medical College Hospital and Research Centre, Chennai, IND

**Keywords:** depression, gerons, historical vignette, indian psychiatry, suicidology, venkoba rao

## Abstract

Dr. Antapur Venkoba Rao, born on August 20, 1927, in Andhra Pradesh, was a pioneering figure in Indian psychiatry, often recognized as the “Father of Indian Psychiatry” and the “Father of Geriatric Mental Health.” His exceptional academic achievements led him to specialize in psychiatry, where he made substantial contributions, particularly in the study of depressive disorders. Dr. Rao's influential research encompassed affective disorders, the clinical application of lithium, and the psychiatry of aging. Among his significant accomplishments, Dr. Rao identified "low melatonin syndrome," linking it to increased relapse rates and suicide risk, and established one of the world’s largest lithium clinics. His work in transcultural psychiatry challenged the prevailing belief that depression was less common in India, revealing how cultural factors can influence suicide risk. As the Head of Psychiatry at Madurai Medical College and Editor-in-Chief of the *Indian Journal of Psychiatry*, Dr. Rao played a crucial role in shaping psychiatric education, research, and practice in India. His impact is further reflected in his numerous awards, including the prestigious Dr. B.C. Roy National Award, and his ongoing engagement in academia and research until his passing on September 25, 2005. Dr. Rao's enduring legacy is evident through his research, publications, and the many psychiatrists he mentored.

## Introduction and background

**Figure 1:**
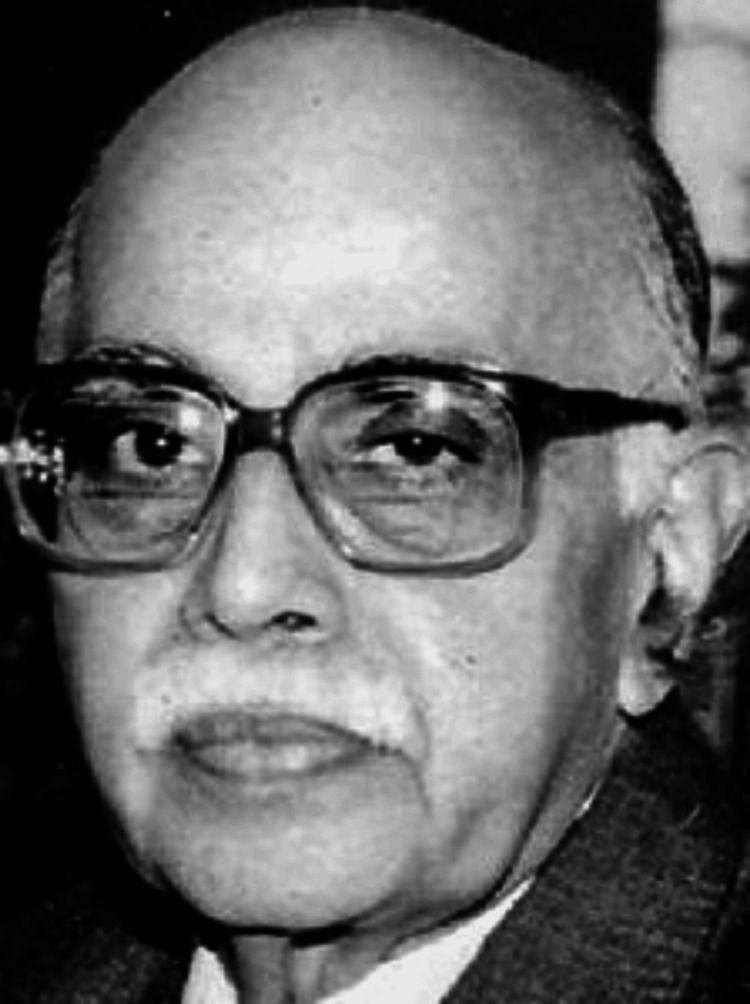
Prof. Dr. Antapur Venkoba Rao (1927-2005) Source: Reference [2] This image is not copyrighted, is in the public domain, and may be freely used for personal or commercial purposes without any restrictions.

This article aims to highlight the substantial contributions of Prof. Dr. Venkoba Rao (Figure 1) to the field of psychiatry. By delving into his research and clinical practice, we aspire to appreciate his enduring legacy. He is often referred to as the “Father of Indian Psychiatry” and “Father of Geriatric Mental Health” [1].

## Review

Early life & education

Antapur Venkoba Rao was born on August 20, 1927, in the village of Kavuttalam, now part of Andhra Pradesh, to Mr. A. Raghavendra Rao and Smt. Lakshmi Devi. As the eldest of eight siblings, including six brothers and two sisters, he spent much of his childhood under the influence of his grandfather, a strict disciplinarian who valued honesty and religious devotion. Venkoba Rao received his early education at Municipal High School in Bellary, where he was a well-regarded student, particularly interested in botany, zoology, and agriculture-fields that aligned with his family's occupation. He pursued his Intermediate studies at Ceded District College in Anantapur, where he excelled, earning first prize in natural sciences and graduating with first-class distinction in physics, chemistry, and natural sciences. Venkoba Rao then attended Madras Medical College for his MBBS, where he received several awards in medicine, obstetrics, and gynecology. Under the mentorship of Dr. S.T. Achar, he gained specialized training in pediatrics. His medical career was further shaped by the guidance of esteemed professionals such as Dr. R.V. Rajam, Dr. J.A.S. Masilamani, Dr. K.S. Sanjivi, Dr. Mohan Rao, and Dr. Ratnavel Subramanian. Venkoba Rao earned his MD in General Medicine and a DPM in Psychiatry from NIMHANS, Bangalore, and later was conferred a PhD and DSc by Madras University.

Personal life

Professor Rao married Dr. Parvathi Devi on February 26, 1950. Dr. Parvathi was an exceptional student, earning the blue ribbon for being the best outgoing student from Madras Medical College. Later she went on to hold prestigious positions as the Director of the Institute of Physiology at Madurai Medical College and Madras Medical College, as well as at Government Rajaji Hospital in Madurai. Her academic interests include neuroendocrinology, neurophysiology, and reproductive physiology. She also published extensively in both national and international journals. The couple was blessed with a daughter and a son. Dr. A.V. Santhi, their daughter, earned her Master in Pathology and had worked at the Institute of Pathology, Institute of Child Health, in Egmore, Chennai. She was married to Dr. S. Ramanathan, a Professor of Pediatric Surgery, and they had a son. Prof. Rao’s son, Dr. A.V. Ashok, serves as an English Professor at the Central Institute of English and Foreign Languages. Mrs. Savitri, his wife, holds a Master's in English Literature and has a daughter.

Career and prestigious positions

Dr. Rao's career began at the Madras Mental Hospital in Kilpauk in 1954, and then, he joined Madurai Medical College. He initially served as an Assistant Professor of Psychiatry at Stanley Medical College and Madras Medical College in Chennai. Following that, from 1962 to 1985, he held the position of Professor and Head of the Department of Psychiatry at Madurai Medical College. Even after retiring, he continued his association as an Emeritus Professor in Madurai Medical College. In addition to his academic roles, Dr. Rao was the Officer-in-Charge of the Indian Council of Medical Research (ICMR) Centre for Advanced Research in Health and Behaviour at Rajaji Government Hospital and Madurai Medical College, Madurai. He also spent time as a visiting scientist at the National Institute of Mental Health in Bethesda, USA. Dr. Rao held a position as an Editor-in-Chief from 1970 to 1978 of the *Indian Journal of Psychiatry* and was a member of the editorial boards of several journals, including the *Indian Journal of Psychiatry*, *NIMHANS*, *Social Psychiatry*, *Transcultural Psychiatric Review*, and *Crisis*. His involvement extended to key roles in organizations such as the ICMR, the National Academy of Medical Sciences (NAMS), the Department of Science and Technology (DST), and the Medical Council of India (MCI). He was also the founder and president of both the Indian Association of Psychiatry and the Indian Association of Suicidology. Throughout his career, Dr. Rao made significant contributions to the field of psychiatry, authoring around 400 national and international publications and a dozen books, including the notable work “Psychiatry of Old Age in India.”

Major contributions to psychiatry research in India

Dr. Rao was a trailblazer in studying the clinical profile of depressive disorders within the Indian context, examining aspects such as epidemiology, genetic factors, long-term progression, and outcomes. He also analyzed the phenomenology of these illnesses through the lens of cultural and philosophical perspectives. His research on suicidal behavior included investigating its etiological factors and developing strategies for prevention while exploring the links between depressive disorders, suicidal tendencies, and pineal hormones. Over the years, Dr. Rao made extensive efforts in exploring various facets of depressive disorder, which was demonstrated in his numerous publications. Renowned as the “Father of Geriatric Mental Health,” he developed a model for health care delivery tailored to the rural elderly population and focused on addressing psychiatric conditions among older adults [2].

Major Contributions to Research on Depressive Illness

His extensive research on depressive illness, carried out over 30 years, involved various significant domains:

Neurobiological domain: He introduced the concept of "low melatonin syndrome," identifying a subtype of depression characterized by reduced melatonin levels, which were associated with increased relapse rates and higher suicidal risk [3,4].

Psychopharmacological domain: He pioneered a lithium clinic that became one of the largest in the world, managing 200 patients with affective disorders. His in-depth investigations into lithium's clinical effects, safety, and mechanisms of action included detailed studies on the dosage, serum levels, and their impact on thyroid function, electrolytes, renal structure and function, memory, and ECG. His work established the efficacy of long-term lithium prophylaxis [5-7].

Experimental domain: His studies covered the effects of psychotropic medications, tricyclic antidepressants, and secretion of melatonin. Notably, he examined "Pineal Response to Lithium" and identified a phenomenon known as the “Tilak effect” [8].

Neuropsychiatric domain: His research on general paresis of the insane (GPI) provided insights into its incidence, serology, clinical types, and treatment outcomes. He was instrumental in demonstrating the presence of *Treponema pallidum* in the frontal lobes of affected patients and its eradication following treatment [9].

Transcultural and philosophical domain: He asserted that ancient Indian knowledge encompassed an understanding of mental health and related disorders from early times. He said, "Undated Indian scriptures written even before the days of the Ramayana or the Mahabharata dealt at length with mental and spiritual health and neural disorders." He explored the deep connection between the Bhagavad Gita and mental health. Viewing the Gita as a profound work of psychotherapy, he introduced a novel perspective to the field of psychotherapy in India [10,11].

Other influential contributions

Suicidology

Dr. Venkoba Rao's work on suicide remains a cornerstone in the understanding and prevention of this tragic outcome. Suicide, particularly among students, has been a critical area of concern, as highlighted in the article. The complex interplay of academic pressures, mental health issues, and lack of adequate support systems has made this demographic particularly vulnerable. Dr. Rao's research delved into the psychological and social factors contributing to suicide, emphasizing the need for a multifaceted approach to prevention. He recognized that the act of suicide is often a cry for help, reflecting deep psychological distress rather than a genuine desire to end life. His work underscored the importance of early identification of mental health issues, proper counseling, and the establishment of robust support systems within educational institutions. Dr. Rao championed increased awareness and the reduction of stigma surrounding mental health issues, especially concerning the intense pressures experienced by students. His contributions have been instrumental in shaping policies and practices aimed at suicide prevention. By addressing the underlying mental health conditions and promoting a supportive environment, Dr. Rao's legacy continues to save lives and guide current practices in psychiatry and mental health [12].

Geriatric Psychiatry

Dr. A. Venkoba Rao made significant contributions to geriatric psychiatry, emphasizing the multifaceted nature of aging and the importance of preventive geriatrics. He described the "universe of gerons" as a diverse group of individuals aged 60 and above, comparing their experience to traveling in an alien country where foreknowledge of its inhabitants and their ways is essential. As life expectancy increases globally, Rao emphasized the dual aspects of aging: on the one hand, it is seen as a phase of wisdom and dignity, while on the other, it is perceived as a period of decline and loss. Rao stressed the importance of health and well-being in aging, noting that only a small proportion of the elderly experience a sense of well-being, with high morbidity rates in India. He emphasized the concept of "compression of morbidity," advocating for a lifestyle that promotes health in old age, which includes a nutritious diet, physical and cognitive activity, and spiritual practices. Rao also discussed the challenges of aging, including depression and the misconception that suicidal thoughts are a natural part of aging. He argued for the importance of understanding and treating these issues to ensure a quality life for the elderly. His work underscores the need for a comprehensive approach to aging that considers physical, mental, and spiritual health. Rao was pivotal in advancing the incorporation of geriatric psychiatry into conventional mental health services in India. He advocated strongly for enhancing mental health care for the elderly, arguing for the development of community-based mental health services that could cater to the needs of older adults, especially in rural areas where such services were scarce. His contributions to aging and geriatric psychiatry were recognized internationally, and he became a respected figure in the global psychiatric community for his work in this field. Through his research, advocacy, and clinical practice, Dr. Venkoba Rao played a crucial role in establishing geriatric psychiatry as an essential discipline in India, ensuring that the mental health needs of the elderly received the attention and care they deserved [13].

Late Life

In his later years, Dr. Venkoba Rao remained engaged and active. He had a keen interest in physics, chemistry, and metallurgy and would attend those lectures, though uncommon for a medical professional, which continued even at the age of 77. He passed away due to acute renal failure at a private hospital on September 25, 2005. His significant contributions to the field of psychiatry will be fondly remembered [14].

Career highlights

Positions Held

Prof. Dr. A. Venkoba Rao held several distinguished positions throughout his career, including serving as a Professor and Head of the Department of Psychiatry at Madurai Medical College from 1962 to 1985. He was also a WHO Consultant on Psychiatric Diseases in 1980 and a Visiting Scientist at the National Institute of Mental Health in the USA in 1986. From 1985 to 1992, he was Officer-in-Charge of the ICMR Centre for Advanced Research on Health and Behaviour in Madurai, and he continued his contributions to the field as Emeritus Professor of Psychiatry from 1985 to 2005.

Editorial Roles

Prof. Dr. A. Venkoba Rao played a significant role in advancing psychiatric knowledge and research in India, serving as the Editor of the *Indian Journal of Psychiatry* from 1971 to 1977. During his tenure, he contributed to shaping the direction of the journal, ensuring the publication of high-quality research, and fostering dialog within the psychiatric community.

Research Contributions

Dr. Venkoba Rao's research spanned multiple key areas in psychiatry, making notable contributions to each. He significantly advanced the understanding and treatment of affective disorders, particularly in depressive diseases. As a pioneer in geriatric psychiatry, he laid the foundation for psychiatric care in the elderly. His work in suicidology provided essential insights into the science of suicide prevention and crisis intervention. In the realm of biological psychiatry, Dr. Rao conducted groundbreaking studies on melatonin in depression and explored the clinical applications of lithium. Additionally, he delved into psychotherapy, drawing inspiration from the Gita and other Indian scriptures to develop culturally relevant therapeutic approaches. His diverse research left a lasting impact on the field of psychiatry.

Dr. Rao explored the unique presentations of depressive symptoms in Indian patients, focusing on guilt and its cultural nuances, particularly in the context of karma and religious beliefs. He examined the divergence in guilt experiences between Indian and Western patients, demonstrating how cultural backgrounds shape emotional and psychological manifestations. His research methodologies included clinical observations and psychological assessments, contributing significantly to cultural psychiatry. Overall, Dr. Rao's insights helped bridge cultural gaps in understanding and treating depression in India [15].

Dr. Venkoba Rao examines the psychological, behavioral, and clinical aspects of HIV/AIDS among Indian patients. He compares 85 HIV-positive individuals to a control group, both from STD clinic attendees, finding a higher rate of promiscuity among female HIV-positive patients but no significant differences in psychiatric illness profiles between the groups. The study explores the impact of HIV disclosure on patients, noting varied psychological responses such as depression, anxiety, and occasionally suicidal ideation. Physical comorbidities like tuberculosis were more prevalent among HIV patients, reflecting unique health challenges in this demographic. Dr. Rao's research underscores the need for targeted mental health support for HIV patients within the Indian health care context [16].

Publications and Books

Dr. Venkoba Rao was a prolific author and coauthor of several influential texts in psychiatry. His works include "Depressive Disease," published under the ICMR, which contributed to the understanding of depression in India. He also authored "Lithium," shedding light on the therapeutic use of lithium in psychiatric practice, and "Psychiatry of Old Age in India," a pioneering work that addressed the unique mental health needs of the elderly in the Indian context. These publications have been instrumental in advancing psychiatric knowledge and practice both nationally and internationally.

Awards and Honors

Dr. Venkoba Rao was the recipient of numerous prestigious awards and honors throughout his distinguished career in psychiatry. He was recognized multiple times by the Indian Psychiatric Society, receiving the "JC Marfatia Award" in 1972, 1975, and 1979. His contributions were further acknowledged with the "Sandoz Award" in 1974, the "PN Raju Oration Award" in 1975, and the "DLN Murti Rao Oration Award" in 1980. Dr. Rao was honored with the "Dr. BC Roy National Award" in 1981, one of the highest accolades in Indian medicine. His recognition continued with the "MN Sen Oration Award" and the "Sir Shriram Memorial Oration Award" in 1983, followed by the "Sandoz Oration Award" at the University of Edinburgh in 1984. He also received the "Academy of Medical Specialities Award" in 1989-1990, the "May & Baker Oration Award" in 1990, the "Dr. NN De Oration Award" in 1991, and the "RV Rajam Oration Award" in 1991-1992. These awards highlight his exceptional contributions to psychiatry, both nationally and internationally.

Fellowships and Memberships

Dr. Venkoba Rao was a distinguished member of several prominent professional bodies, both in India and internationally. He was a "Fellow of the National Academy of Medical Sciences (India)" and a "Fellow of the Royal College of Psychiatrists, London." His global influence in psychiatry extended further, as he was a "Member of the American Psychiatric Association," a "Fellow of the Royal Australian and New Zealand College of Psychiatrists," and a "Member of the Canadian Association of Psychiatrists." Additionally, he was a "Fellow of the Indian Academy of Sciences, Bangalore," a "Life Fellow of the Indian Psychiatric Society," and a "Fellow of the Indian Academy of Neurology," reflecting his deep involvement in advancing mental health across various platforms.

Other contributions

In addition to his academic and clinical contributions, Dr. Rao played a significant role in broader societal initiatives. He served on the Tamil Nadu Prison Reform Commission, contributing to improving mental health care in the prison system. He was also a member of the National Committee to Study the Extent of Drug Addiction in India in 1976, reflecting his commitment to tackling critical public health issues. Moreover, he actively engaged with the World Psychiatric Association, further influencing the global psychiatric community.

## Conclusions

In conclusion, the life and work of Dr. Venkoba Rao stand as a remarkable testament to his pioneering spirit and unwavering dedication to psychiatry. His research on suicidal behavior provided critical insights into the interplay of psychological, biological, and cultural factors, laying the groundwork for modern approaches to suicide prevention. As the Father of Geriatric Psychiatry, Dr. Rao not only highlighted the unique mental health needs of the elderly but also introduced comprehensive care models, ensuring dignity and well-being in aging populations. Furthermore, his integration of psychotherapy with Indian philosophical thought, particularly through the teachings of the Bhagavad Gita, demonstrated his innovative approach to mental health, bridging cultural and clinical paradigms.

Dr. Rao's legacy continues to influence psychiatric research, education, and clinical practices globally, inspiring future generations to advance mental health care with compassion and scientific rigor. By honoring his contributions, we reaffirm the importance of his vision in shaping the field of psychiatry today and beyond.

## References

[1] Katram B (2016). Dr. Antapur Venkoba Rao. Telangana J Psychiatry.

[2] Dr. Antapur Venkoba Rao Venkoba Rao. Indian National Science Academy.

[3] Rao AV (1984). Depressive illness in India. Indian J Psychiatry.

[4] Rao AV, Devi SP, Srinivasan V (1983). Urinary melatonin in depression. Indian J Psychiatry.

[5] Rao AV, Hariharasubramanian N (1980). Electrocardiographic changes during lithium treatment. Indian J Psychiatry.

[6] Rao AV, Sugumar A, Hariharasubramanian N, Shanti AV, Ramachandran K, Kumar CL (1981). Lithium and kidney: (a study of renal biopsy in lithium patients). Indian J Psychiatry.

[7] Rao AV, Hariharasubramanian N, Devi SP, Sugumar A, Srinivasan V (1982). Lithium prophylaxis in affective disorder. Indian J Psychiatry.

[8] Devi SP, Rao AV (1982). Pineal response to lithium. Indian J Psychiatry.

[9] Rao AV, Ranganathan PS, Natarajan M (1972). General paresis in the psychiatric department of a general hospital in India. Br J Psychiatry.

[10] Rao AV (2002). 'Mind' in Indian philosophy. Indian J Psychiatry.

[11] Rao AV (1980). Gita and mental sciences. Indian J Psychiatry.

[12] Rao VA, Chinnian RR (1972). Attempted suicide and suicide among ‘student’ in Madurai. Indian J Psychiatry.

[13] Rao AV, Madhavan T (1982). Gerospsychiatric morbidity survey in a semi-urban area near Madurai. Indian J Psychiatry.

[14] Singh A (2005). Dr. A. Venkoba Rao. Mens Sana Monogr.

[15] Venkoba Rao A (2009). Depressive illness and guilt in Indian culture. Asian J Psychiatr.

[16] Rao AV, Swaminathan R, Venkataram MK, Ramajayam S, Parhee R, Kumar N, Luthra UK (1991). A clinical and behavioural study of HIV infected subjects-a comparison with STD subjects. Indian J Psychiatry.

